# Application of multi-label classification models for the diagnosis of diabetic complications

**DOI:** 10.1186/s12911-021-01525-7

**Published:** 2021-06-07

**Authors:** Liang Zhou, Xiaoyuan Zheng, Di Yang, Ying Wang, Xuesong Bai, Xinhua Ye

**Affiliations:** 1 Department of Endocrinology, Changzhou No.2 People’s Hospital Affiliated to Nanjing Medical University, 29 Xinglongxiang Road, Changzhou City, 213000 Jiangsu Province China; 2 Shanghai Jiao Tong University School of Medicine, Shanghai, 200025 China; 3 Capital Medical University, Beijing, 100053 China

**Keywords:** Diabetic complication, Machine learning, Multi-label classification, Correlation, Key indicators

## Abstract

**Background:**

Early diagnosis for the diabetes complications is clinically demanding with great significancy. Regarding the complexity of diabetes complications, we applied a multi-label classification (MLC) model to predict four diabetic complications simultaneously using data in the modern electronic health records (EHRs), and leveraged the correlations between the complications to further improve the prediction accuracy.

**Methods:**

We obtained the demographic characteristics and laboratory data from the EHRs for patients admitted to Changzhou No. 2 People’s Hospital, the affiliated hospital of Nanjing Medical University in China from May 2013 to June 2020. The data included 93 biochemical indicators and 9,765 patients. We used the Pearson correlation coefficient (PCC) to analyze the correlations between different diabetic complications from a statistical perspective. We used an MLC model, based on the Random Forest (RF) technique, to leverage these correlations and predict four complications simultaneously. We explored four different MLC models; a Label Power Set (LP), Classifier Chains (CC), Ensemble Classifier Chains (ECC), and Calibrated Label Ranking (CLR). We used traditional Binary Relevance (BR) as a comparison. We used 11 different performance metrics and the area under the receiver operating characteristic curve (AUROC) to evaluate these models. We analyzed the weights of the learned model and illustrated (1) the top 10 key indicators of different complications and (2) the correlations between different diabetic complications.

**Results:**

The MLC models including CC, ECC and CLR outperformed the traditional BR method in most performance metrics; the ECC models performed the best in Hamming loss (0.1760), Accuracy (0.7020), F1_Score (0.7855), Precision (0.8649), F1_micro (0.8078), F1_macro (0.7773), Recall_micro (0.8631), Recall_macro (0.8009), and AUROC (0.8231). The two diabetic complication correlation matrices drawn from the PCC analysis and the MLC models were consistent with each other and indicated that the complications correlated to different extents. The top 10 key indicators given by the model are valuable in medical application.

**Conclusions:**

Our MLC model can effectively utilize the potential correlation between different diabetic complications to further improve the prediction accuracy. This model should be explored further in other complex diseases with multiple complications.

**Supplementary Information:**

The online version contains supplementary material available at 10.1186/s12911-021-01525-7.

## Introduction

Complications of diabetes are the leading cause of death in diabetic patients [1], with 76.4% of diabetic patients reporting at least one complication [2]. These include cardio- or cerebrovascular diseases, neuropathy, nephropathy, retinopathy, and foot disease [3], which compromise patient quality of life and bring an economic burden to the healthcare system [4]. Therefore, how to quickly and accurately diagnose and analyze diabetic complications is a topic worthy studying. Modern electronic health records (EHRs) [5, 6] is a rich resource for clinical data from which newer physical indicators can be identified as predictors of diabetic complications to assist in treatment planning. However, comprehensive analysis of such data remains a challenge.

Machine learning has great advantages when dealing with massive data with both high dimensional attributes and tremendous number of instances, which has been widely applied in disease prediction [7]. The binary classification model is typically adopted by most approaches [8–14] and shows promising results for the prediction of diabetic complications. However, each diabetic complication was modeled and predicted independently in these studies, making it impossible to leverage the potential correlations among diabetes complications.

Multi-label classification (MLC) models have shown great promise in text categorization, image classification, automatic annotation for multimedia content, bioinformatics, web mining, rule mining, information retrieval, tag recommendation, and other diverse fields [15].By modeling multiple labels simultaneously, multi-label learning can identify correlations among different labels more easily. Therefore, we sought to apply an MLC model to leverage the correlations among diabetic complications and further improve their predictive performance.

### Related work

Compared to traditional medical studies with a hypothesis-driven perspective, [16–18] data mining based on data features is better suited for studying nonlinear interactions in large diabetes data sets and can more-accurately assess and predict disease risk. For predicting the outcomes of diabetes-related complications and death, the Risk Equations for Complications of Type 2 Diabetes equations [19, 20], were derived from the Action to Control Cardiovascular Risk in Diabetes randomized trial [21] and the UK Prospective Diabetes Study Outcomes Model 2 [22] that used data of 3,642 patients from the United Kingdom Prospective Diabetes Study. These Cox proportional hazards models have good discrimination and calibration, and they are used widely. However, they were developed based on clinical trial data with limited case numbers and potential study-specific biases. Conversely, machine learning predicts diabetes complications by using EHRs, a preferable data set with an abundance of valuable information describing a patient’s healthcare experience.

While the machine learning models [8–14]are widely used in predictions of diabetic complications, a more suitable method, multi-label learning, has been used rarely. Single-label methods predict diabetes complications separately based on whether or not one complication occurs. MLC is a machine learning method used in many clinical applications. A chronic disease risk dataset containing 110,300 patients, 62 symptoms, and six disease labels (hypertension, diabetes, fatty liver, cholecystitis, heart disease, and obesity) was transformed into a multiclass classification, and the MLC classifiers proved to have good performance [23]. To explore a diagnostic method that classifies and evaluates each psychotic disorder simultaneously, Folorunso et al. used an MLC and demonstrated that the MLC was more efficient than a previous study [24]. In the diabetes field, MLC has been applied to label the retinal image of normal/abnormal regions, patient age, ethnicity, race, and diabetic type for diabetic retinopathy differentiation and can improve retinal image classification [25].

Diabetic complications are highly related. Retinal vascular trait changes have been linked with cardiovascular disease [26, 27]and stroke [28] and structural variations in retinal vasculature can predict cardiovascular risk [29–31]. Additionally, Xu et al. found that microalbuminuria in type 2 diabetes is associated with both retinal vascular caliber and geometry, and that retinal and renal microvasculature share similar pathophysiological changes during early diabetes due to abnormal glucose metabolism and other processes [32]. Interactions among diabetes complications need to be identified and applied to facilitate early diagnoses. Since diabetes patients may suffer from multiple diabetic complications, the diagnosis of complications (labels) using indicators (attributes) is an MLC problem. These class/label variables usually exhibit conditionally dependent relationships among themselves, which must be modeled and learned. To frame the diabetic complication classification into an MLC problem, multi-label methods were first applied to predict diabetes complications from EHRs by Bai et al. The results indicated that random k-label sets and chained classifiers performed better than binary relevance, least combination, and pruned sets [33].We aimed to identify the best MLC model to predict diabetic complications and inform clinical decisions that could help personalize type 2 diabetes management.

### Objective

Traditional methods utilized the binary classification model to predict each diabetic complication independently and failed to leverage the potential correlations among diabetic complications. Through statistical analysis of the data in EHRs, we aimed to: (1) find correlations between different diabetic complications and (2) leverage these correlations to improve the predictive performance via an MLC model.

## Material and methods

### Data source

The study used demographic characteristics and laboratory data obtained from EHRs data, from May 2013 to June 2020, for patients admitted to Changzhou No.2 People’s Hospital, the affiliated hospital of Nanjing Medical University in China. This study was approved by the ethics committee of Changzhou No.2 People’s Hospital, the affiliated hospital of Nanjing Medical University. A total of 9765 adult patients with diabetes, were eligible for this study. The demographic variables of patients with diabetes are summarized in Table 1. Due to the absence of BMI values for some patients, Table 1 only displays BMI characteristics for 8,831 patients.

**Table 1:** Overview of demographic characteristics

Demographic characteristics (labels)	Diabetes without complications	Retinopathy	Nephropathy	Neuropathy	Peripheral vascular disease	All
Total patients	288	5129	1851	7872	5519	9765
*Age, years (%)*						
< 40	33 (11.5)	313 (6.1)	112 (6.1)	343 (4.4)	75 (1.4)	544 (5.6)
40–49	62 (21.5)	703 (13.7)	214 (11.6)	905 (11.5)	473 (8.6)	1247 (12.8)
50–59	103 (35.8)	1438 (28.0)	460 (24.9)	2038 (25.9)	1371 (24.8)	2600 (26.6)
≥ 60	90 (31.3)	2675 (52.2)	1065 (57.5)	4586 (58.3)	3600 (65.2)	5374 (55.0)
*Gender (%)*						
Male	141 (49.0)	2803 (54.7)	1075 (58.1)	4352 (55.3)	2348 (42.5)	4438 (45.4)
Female	147 (51.0)	2326 (45.3)	776 (41.9)	3520 (44.7)	3171 (57.5)	5327 (54.6)
*BMI*						
BMI (± SD) (kg/m^2^)	35.4 (23.62–47.18)	24.4 (19.74–29.06)	24.1 (20.38–27.82)	27.06 (26.63–27.49)	26.7 (24.6–26.8)	35.2 (23.2–47.2)
BMIcategory (kg/m^2^) (%)	n = 252	n = 4789	n = 1633	n = 7133	n = 5037	n = 8831
< 18.5	2 (0.8)	147 (3.1)	67 (4.1)	247 (3.5)	135 (2.7)	296 (3.4)
18.5–25	104 (41.3)	2565 (53.6)	746 (45.7)	3739 (52.4)	2598 (51.6)	4612 (52.2)
25–30	103 (40.9)	1761 (36.8)	692 (42.4)	2661 (37.3)	1969 (39.1)	3305 (37.4)
30–34.9	30 (11.9)	273 (5.7)	107 (6.6)	411 (5.8)	286 (5.7)	503 (5.7)
≥ 35	13 (5.2)	43 (0.9)	21 (1.3)	75 (1.1)	49 (1.0)	115 (1.3)

BMI values were unavailable for some patients; hence, BMI values are given for a total of 8831 patients only

### Data preprocessing

There are total 141 indicators in the raw data of EHRs. However, there are 47 indicators that are missing in more than 95% patients. These indicators can be seen as useless, therefore we deleted them in our study. Besides, we combined the height and weight to be BMI, and finally 93 clinical parameters collected from EHRs are considered as possible risk factors (see Additional file 1: Table 1). The data assignment methods are shown in Additional file 1: Table 2. Besides, for indicators which have few missing values in some patients, we adopt the mean value and mode to fill the continuous and discrete indicators, respectively.

### Multi-label classification models

In general, multi-label classification algorithms can be divided into two categories: problem transformation methods and algorithm adaptation methods [15]. As the name suggests, problem transformation methods tackle the multi-label classification problem by transforming it into other well-established learning problems. Representative algorithms include first-order approach Binary Relevance (BR) [34] and high-order approach Classifier Chains (CC) [35, 36] which transform the task of multi-label learning into the task of binary classification, second-order approach Calibrated Label Ranking (CLR) [37] which transforms the task of multi-label learning into the task of label ranking, and high-order approaches Random k-Label Sets (RAkEL) and Label Power Set (LP) [38] which transform the task of multi-label learning into the task of multi-class classification. Different from problem transformation methods, algorithm adaptation methods tackle multi-label learning problem by adapting some existing learning algorithms to the multi-label learning scenario directly. Representative algorithms include first-order approach Multi-Label k-Nearest Neighbor (ML-kNN) [39] which adapts the k-nearest neighbor learning algorithms, Multi-Label Decision Tree (ML-DT) [40] which adapts decision tree techniques, second-order approach Ranking Support Vector Machine (Rank-SVM) [41] which adapts kernel techniques, and second-order approach Collective Multi-Label Classifier (CML) [42] which adapts information-theoretic techniques. In this paper, we compare some representative methods which are detailly introduced in the following sections.

### Binary relevance

The basic idea of binary relevance is to decompose the multi-label classification problem into multiple independent binary classification problems, where each binary classification problem corresponds to a possible label in the label space [34]. For class j, binary relevance method first constructs a binary training set by the following metric:

$$\begin{aligned} & {\mathcal{D}}_{j} = \left\{ {\left( {x_{i} ,\phi \left( {Y_{i} ,y_{j} } \right)} \right)\left| {1 \le i \le m} \right.} \right\} \\ { } & {\text{where }}\,\phi \left( {Y_{i} ,y_{j} } \right) = \left\{ {\begin{array}{*{20}c} { + 1,} & {{\text{if }}\,y_{j} \in Y_{i} } \\ { - 1,} & {{\text{otherwise}}} \\ \end{array} .} \right. \\ \end{aligned}$$

Then, a binary classifier for class j is built using existing binary classification algorithms to justify if the instance belongs to class j. Finally, total Q binary classifiers will be built where Q is the number of all possible classes. Therefore, in multi-label classification learning, the instance will be classified by Q binary classifiers. The final classification result for an instance is the combination of all binary classifiers.

### Label power set

This method takes each unique subset (distinct label set) of labels that exists in multi-label dataset as a single label. Therefore, label power set introduces new sets of labels and transforms the multi-label classification into a multi-class classification task [38]. For each test instance, the classifier of label power set predicts it into one label which is originally a set of multiple labels in the multi-label dataset. Label power set is simple but it may introduce some even many classes which only have a few samples, and further aggravate the class imbalance.

### Classifier chains

The basic idea of classifier chains is to transform the multi-label learning problem into a chain of binary classification problems, where subsequent binary classifiers in the chain are built upon the predictions of previous ones [35, 36]. For example, when building the j-th binary classifier, the input of the model will be

$$x_{i}^{*} = [x_{i} ,p_{1} ,p_{2} ,...,p_{j - 1} ]$$

where $x_{i}$ is the original feature, $p_{k}$ is the prediction of the kth binary classifier, and $x_{i}^{*}$ is the concatenation of $x_{i}$ and $p_{1} ,p_{2} ,...,p_{j - 1}$. Then, the jth binary classifier will be built on this concatenated feature and produce the probability of this sample belonging to the jth class. For test instances, the associated label set is predicted by traversing the classifier chain iteratively. It is obvious that, for the classifier chain method, its effectiveness will be largely affected by the ordering of classes during building the classifier chains. Besides, classifier chains has the advantage of exploiting the label correlations while loses the opportunity of parallel implementation due to the chaining property [35, 36].

### Calibrated label ranking

The basic idea of this algorithm is to transform the multi-label learning problem into the label ranking problem, where ranking among labels is fulfilled by techniques of pairwise comparison. For multi-label learning problem with Q classes, a total of Q(Q-1)/2 binary classifiers will be constructed, one for each label pair [37]. In detail, for each pair, we first construct a corresponding binary training set, where each instance has distinct relevance to the two labels in the label pair and only belongs to one label in the label pair. Then, some binary learning algorithms will be utilized to induce a binary classifier for each label pair. For testing, each instance will first be fed into the Q(Q−1)/2 learned binary classifiers to obtain the overall votes on each possible class label. And then the labels can be ranked according to their obtained votes.

### Model evaluation metrics

In the literature, there is no generally adopted metrics for evaluating multi-label classification models. Therefore, several measures from multi-class classification and information retrieval were usually adopted and adapted to measure multi-label classification performance [43]. In our experiments, we used various evaluation measures that have been suggested by previous studies [43], which are defined in Table 2. The evaluation metrics can be divided into two types: example-based metrics and label-based metrics. In Table 2, $y_{i}$ denotes the set of true labels of example $x_{i}$; $h\left( {x_{i} } \right)$ denotes the set of predicted labels for the same sample; $\Delta$ stands for the symmetric difference between the two sets; N is the number of examples; Q is the total number of possible class labels; $tp_{j}$ is the number of true positive for label $j$; $fp_{j}$ is the number of false positive for label $j$; $p_{j}$ and $r_{j}$ are the precision and recall for class $j$. It should be noted that, the accuracy and subset accuracy used here for multi-label classification are example-based measures and computed on the label set, which are different from the general accuracy used in binary or multi-class classification. Besides, the macro average metrics compute the metric individually for each class and then take an average, and the micro average metrics aggregate the contributions of all classes to compute the average metric. Following previous studies [40], we use both macro and micro metrics to evaluate the model.

**Table 2:** Evaluation metrics

Metrics	Example-based measures
Hamming Loss	${\text{Hamming - loss}}(h) = \frac{1}{N}\mathop \sum \limits_{i = 1}^{N} \frac{1}{Q}\left| {h\left( {x_{i} } \right)\Delta y_{i} } \right|$
Accuracy	${\text{Accuracy}}(h) = \frac{1}{N}\sum\limits_{i = 1}^{N} {\left| {\frac{{h\left( {x_{i} } \right) \cap y_{i} }}{{h\left( {x_{i} } \right) \cup y_{i} }}} \right|}$
Precision	${\text{Precision}}(h) = \frac{1}{N}\mathop \sum \limits_{i = 1}^{N} \frac{{\left| {h\left( {x_{i} } \right) \cap y_{i} } \right|}}{{\left| {y_{i} } \right|}}$
Recall	${\text{Recall}}(h) = \frac{1}{N}\mathop \sum \limits_{i = 1}^{N} \frac{{\left| {h\left( {x_{i} } \right) \cap y_{i} } \right|}}{{\left| {h\left( {x_{i} } \right)} \right|}}$
F1-score	$F_{1} = \frac{1}{N}\mathop \sum \limits_{i = 1}^{N} \frac{{2 \times \left| {h\left( {x_{i} } \right) \cap y_{i} } \right|}}{{\left| {h\left( {x_{i} } \right)} \right| + \left| {y_{i} } \right|}}$
Subset Accuracy	${\text{Accuracy}}_{{{\text{sub}}}} (h) = \frac{1}{N}\mathop \sum \limits_{i = 1}^{N} I\left( {h\left( {x_{i} } \right) = y_{i} } \right)$
Label-based Measures	
Macro-precision	${\text{Macro - precision}} = \frac{1}{Q}\mathop \sum \limits_{j = 1}^{Q} \frac{{tp_{j} }}{{tp_{j} + fp_{j} }}$
Macro-recall	${\text{Macro - recall}} = \frac{1}{Q}\mathop \sum \limits_{j = 1}^{Q} \frac{{tp_{j} }}{{tp_{j} + fn_{j} }}$
Macro-F1-score	${\text{Macro - F1}} = \frac{1}{Q}\mathop \sum \limits_{j = 1}^{Q} \frac{{2 \times p_{j} \times r_{j} }}{{p_{j} + r_{j} }}$
Micro-precision	${\text{Micro - precision}} = \frac{{\mathop \sum \nolimits_{j = 1}^{Q} tp_{j} }}{{\mathop \sum \nolimits_{j = 1}^{Q} tp_{j} + \mathop \sum \nolimits_{j = 1}^{Q} fp_{j} }}$
Micro-recall	${\text{Micro - recall}} = \frac{{\mathop \sum \nolimits_{j = 1}^{Q} tp_{j} }}{{\mathop \sum \nolimits_{j = 1}^{Q} tp_{j} + \mathop \sum \nolimits_{j = 1}^{Q} fn_{j} }}$
Micro-F1-score	${\text{Micro - F1}} = \frac{{2 * {\text{micro - precision}} * {\text{micro - recall}}}}{{{\text{micro - precision}} + {\text{micro - recall}}}}$

### Correlation analysis

**Fig. 1:**
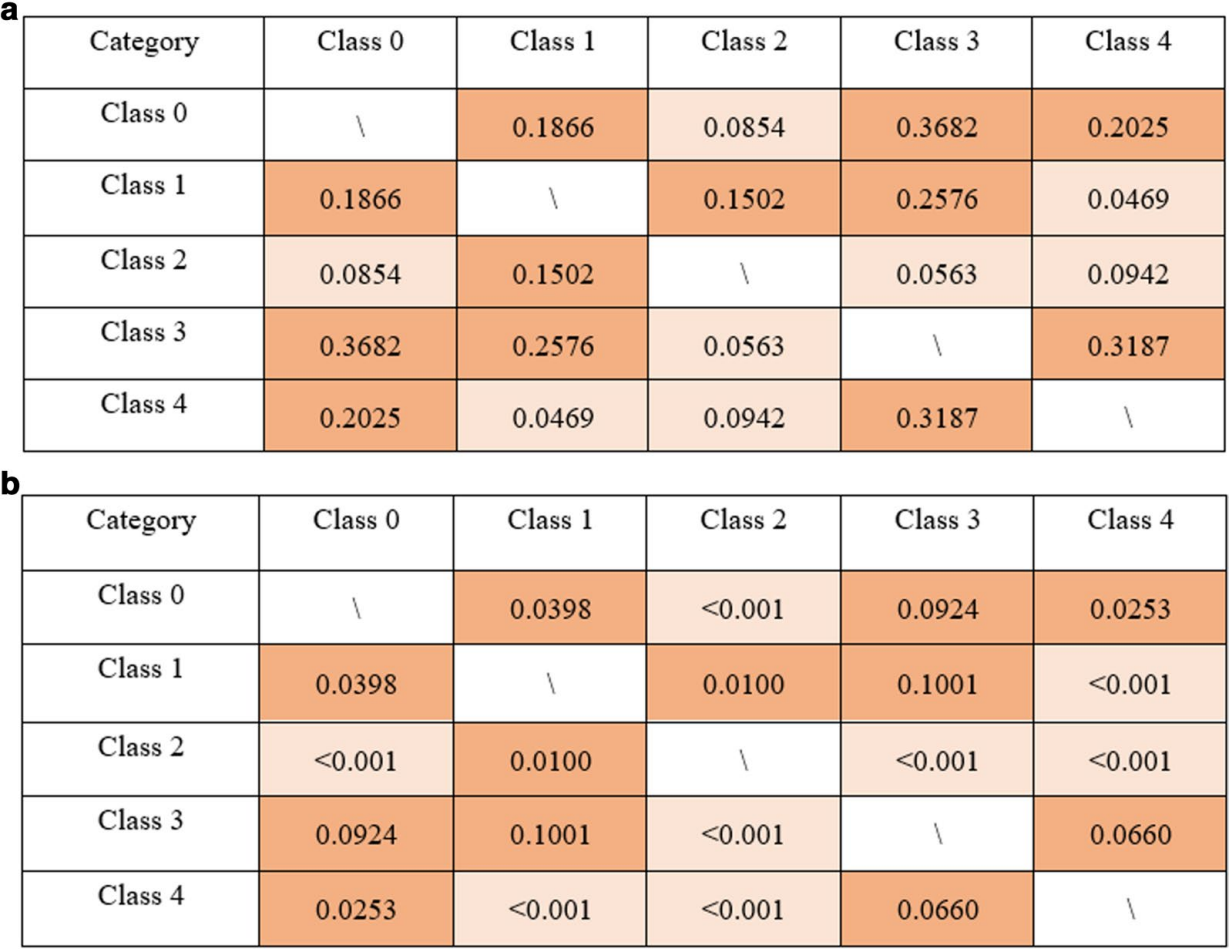
**a** Pearson Correlation Coefficient; **b** Model Correlation Coefficient. Different complications share correlation to different extend. In **a**, a relatively correlation (coefficient > 0.1) is marked in deep orange while a relatively weak correlation (coefficient < 0.1) is marked in light orange. In **b** the coefficient below 0.001 (low correlation) is marked in light orange and the others marked in deep orange

The Pearson correlation coefficient (PCC) is a statistic that measures the linear correlation between two variables X and Y, with values between + 1 and − 1. A value of + 1 is a 100% positive linear correlation, 0 is no linear correlation, and − 1 is a 100% negative linear correlation. Pearson correlation coefficients were calculated using the EHRs raw data to analyze the statistical relationships among different diabetic complications, as shown in Fig. 1a. This motivated the introduction of an MLC model to leverage this correlation toward further predictions.

The relationships among diabetic complications could also be evaluated using our models. In the CC model, the label of the previous class in the chain was used as an input to predict the current class. We used the weight (importance) of the previous class as the correlation coefficient of the two classes. The correlation coefficient matrix obtained in the CC model is presented in Fig. 1b.

The relative strength of both correlation coefficient matrices was surprisingly consistent (Fig. 1a, b), which strongly suggests the validity of our model. Our model further indicates that nephropathy, retinopathy, and peripheral vascular disease are all correlated with each other directly, except for retinopathy with neuropathy.

## Results

We compared the binary classification model with 4 MLC models under 11 different performance evaluation metrics. Then, we leveraged the receiver operating characteristic (ROC) curve and area under the curve (AUC) to further evaluate the performance of different models. Besides, we provided the top 10 key indicators for each complication. Finally, we provided a comparison between the correlation coefficient matrix derived from our model and statistical analysis of the EHRs’ raw data.

### Default experimental setup

For all the experiments, we used classes 0, 1, 2, 3, and 4 to represent five different categories: (0) diabetes without complications, (1) retinopathy, (2) nephropathy, (3) neuropathy and (4) peripheral vascular disease. The whole dataset was randomly split into two disjoint subsets: training set (75%) and test set (25%). Then, five-fold cross validation was employed for all experiments to tune the hyperparameters within the training dataset. As for the base model, we mainly adopted Random Forest technique as the base model to conduct the classification task, which is usually taken as the base model in previous related studies and performed best in compared models [13], and is easy to implement. Besides, we also conducted experiments using XGBoost as the base model, whose results are displayed in Additional materials (see Additional file 1: Table 3 and Fig. 1). In addition, to alleviate the class imbalance problem, we set different weights for different categories according to the proportion of samples belonging to each category.

### Machine learning experiment results

We conducted comparative experiments to evaluate the prediction introduced by the MLC models. The prediction results, shown in Table 3, were evaluated using 11 performance evaluation metrics: hamming loss, accuracy, f1 score, precision, recall, f1 micro, f1 macro, precision micro, precision macro, recall micro, and recall macro. As shown in Table 3, apart from the label power set (LP), all MLC models outperformed the binary classification model in most evaluation performance metrics. Among the four MLC models, the ensemble classifier chains (ECC) yielded the best performance for most metrics. The LP method performed the worst, which may be because it introduced many classes with only a few samples, which aggravated the class imbalance.

**Table 3:** Experiment results on 5 different models including 11 performance evaluation metrics

Metric	Traditional model	The MLC models			
	BR	LP	CC	ECC	CLR
Example-based Metrics					
Hamming loss	0.1864	0.2141	**0.176**	**0.176**	0.1763
Accuracy	0.6816	0.6364	0.6948	**0.702**	0.6875
F1_score	0.7661	0.721	0.7763	**0.7855**	0.7711
Precision	0.8163	0.7706	0.8298	**0.8649**	0.8156
Recall	0.78	0.7406	0.7861	0.7727	**0.792**
Label-based Metrics					
F1_micro	0.789	0.7559	0.8000	**0.8078**	0.7968
F1_macro	0.7593	0.708	0.7714	**0.7773**	0.7631
Precision_micro	0.7709	0.7396	0.7757	0.7592	**0.7863**
Precision_macro	0.7884	0.6822	0.7964	0.7689	**0.8073**
Recall_micro	0.806	0.7731	0.8261	**0.8631**	0.8077
Recall_macro	0.7476	0.7386	0.764	**0.8009**	0.7394

The MLC models included a Label Power Set (LP), Classifier Chains (CC), Ensemble Classifier Chains (ECC), and Calibrated Label Ranking (CLR). The traditional model Binary Relevance (BR) is used as a comparison. The best performing method is in bold

### Analysis of area under the receiver operating characteristic curve

**Fig. 2:**
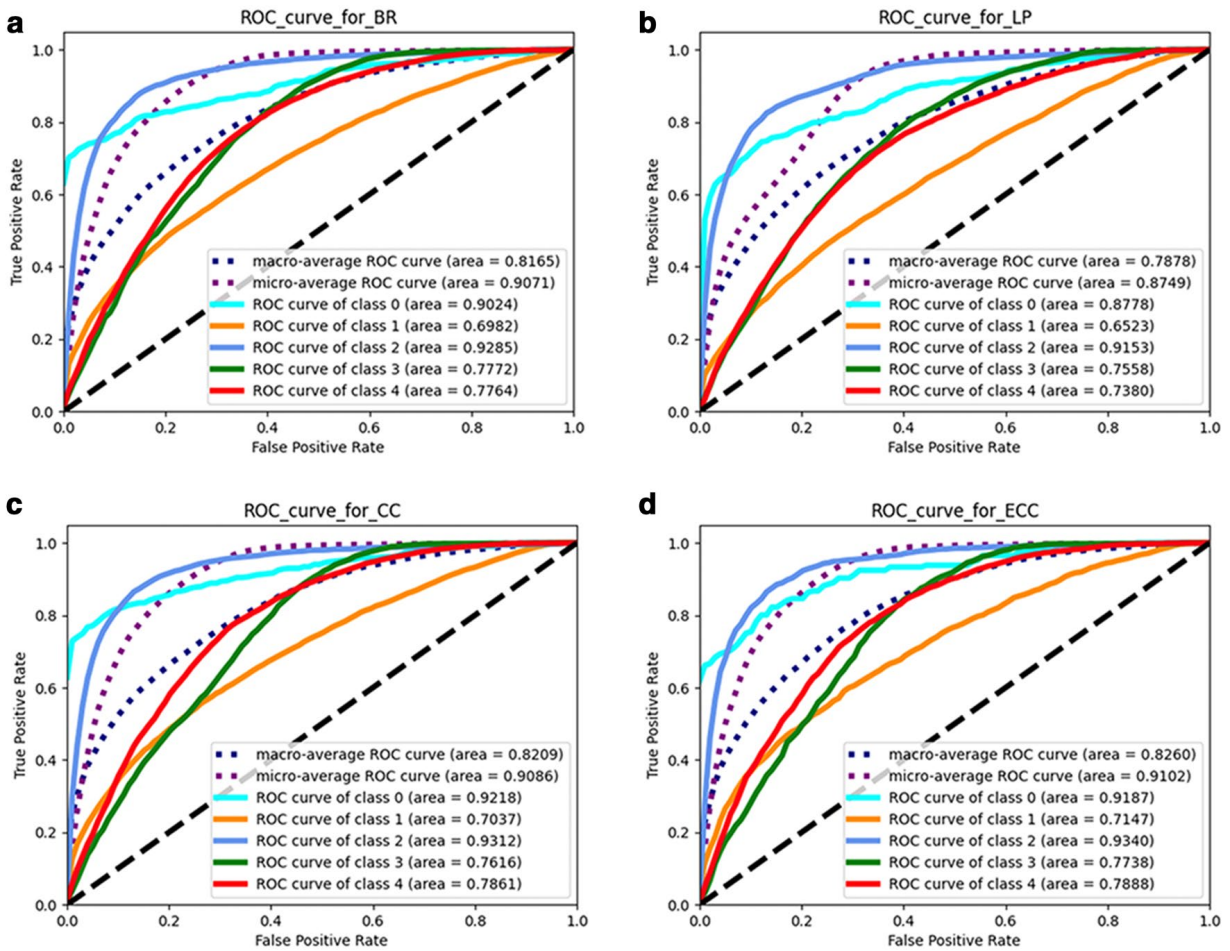
The experimental results of 4 different models, BR, LP, CC, and ECC. In each figure, the ROC curves of different complications are marked by different colors accordingly

We leveraged the AUC and ROC curve to further evaluate the performance of different models for each diabetic complication. The ROC curve is a graphical representation for showing the trade-off between the recall/sensitivity/true positive rate and false-positive rate; the precision-recall curve is a graphical representation for showing the trade-off between precision and recall. The experimental results of four different models, binary relevance (BR), LP, classifier chains (CC), and ECC, are shown in Fig. 2a–d. It should be noted that the area under the receiver operating characteristic curve (AUROC) result for calibrated label ranking (CLR) was not available, as it could not provide the prediction probability. As shown in Fig. 2a–d, the ECC model yielded the highest AUROC, and all the MLC models, except LR, outperformed the BR model. It can be seen that nephropathy (class 2) received the highest AUROC among all diabetic complications, while retinopathy (class 1) yielded a relatively low AUROC.

### Key features analysis

The 10 most correlated indicators given by the learned random forest model for the five categories of diabetic states are listed in Table 4, in which a higher ranking indicates a higher correlation coefficient.

**Table 4:** Top10 key features in prediction

Diabetes without complications	Retinopathy	Nephropathy	Neuropathy	peripheral vascular disease
Age	Urinary mAlb	Urinary mAlb	Urinary mAlb	Age
ALT	CPK	ACR	Urine Cr	Urine Cr
GGT	ESR	Urinary mAlb /Cr	Age	Urinary mAlb
Triglyceride	Age	Urine protein	Serum Ca	Serum P
Albumin	Adenosine deaminase	Serum Cr	Urine RBC	Serum Ca
Glucose	CK-MB	BUN	Crystalluria	Apo A1
Cholesterol	Total CO2	Urine Cr	Serum P	Apo B
Serum Cr	Urinary mAlb / Cr	Uric acid	CPK	Glucose
Urine Cr	INR	Albumin	Hyaline cast	USECs
ALT/AST	Monocyte %	Fibrinogen	Cholinesterase	Serum Cr

*ALT* alanine aminotransferase, *AST* aspartate aminotransferase, *GGT* gamma-glutamyl transferase, *Cr* creatinine, *mAlb* microalbumin, *CPK* creatine phosphokinase, *ESR* erythrocyte sedimentation rate, *CK-MB* creatine kinase-MB, *INR* PT international standardized ratio, *ACR* albumin-to-creatinine ratio, *BUN* blood urea nitrogen, *Ca* calcium, *P* phosphorus, *RBC* red blood cell, *Apo A1* apolipoprotein A1, *ApoB* apolipoprotein B, *USECs* urinary squamous epithelial cells

The majority of indicators were consistent with known predictive factors, for example; age, levels of glucose, serum/urine creatinine (Cr), and lipids (Table 4). Several predictors were consistent with the latest medical research: fibrinogen, plasma albumin, hematocrit for nephropathy [44–47], and adenosine deaminase-2 for retinopathy [44–47]. These results suggest validation of our models. Since this is the first application of an MLC model to the prediction of diabetic complications, new indicators not identified in medical research were identified that suggest future studies for medical verification. For example, creatine phosphokinase (CPK), creatine kinase-MB, erythrocyte sedimentation rate, total carbon dioxide in the blood, international normalized ratio, and monocyte percentage for diabetes retinopathy; urinary microalbumin, urine Cr, serum Calcium (Ca), urine red blood cells, crystalluria, serum phosphorous (P), creatine kinase, and hyaline cast cholinesterase for diabetes neuropathy; and urine Cr, urinary microalbumin, serum P, serum Ca, urinary squamous epithelial cells, and serum Cr for peripheral vascular disease.

## Discussion

To our knowledge, this is the first study to characterize the risk of developing diabetic complications and assess the relationship of these complications in the Chinese population.

This is the first application of an MLC model to leverage the correlations among diabetic complications for better predictive performance. The multi-label model can simultaneously predict multiple factors, including neuropathy, nephropathy, retinopathy, and peripheral vascular disease. This type of multiple-complication screening is widely used in real-world diagnosis, since patients can have multiple simultaneous complications. Most of the MLC models outperformed the BR model, which was used in previous studies. With the MLC model, high predictive performance was achieved, evaluated under multiple evaluation metrics and AUC, as shown in Table 3 and Fig. 2a, b. The ECC model yielded the highest AUROC of 0.826 and led in most of the evaluation metrics. The experimental results further indicate that multi-label models are efficient for leveraging correlations among diabetes complications.

On analyzing the correlation between and the predictive factors of diabetes complications from pathological perspective. The novel factors identified in our models are consistent with recent studies. Adenosine deaminase-2 was identified as a predictor of diabetic retinopathy, consistent with the work of Elsherbiny et al. [48]. It is shown that fibrinogen [44] can be used as a predictor of end-stage renal disease in type 2 diabetes, and plasma albumin [45, 46] can be used as an indicator of the prognosis of renal disease. Robles et al. used hematocrit, urea, and sex to predict the progression of diabetic nephropathy [47]. Issar et al. reported that patients with chronic kidney disease had severe neuropathy phenotypes and shared nerve dysfunction features with CKD [49]. These evidences further proved the validity of our model. In addition, our multi-label model identified several novel predictors that were not found by previous risk assessment methods. For example, CPK, and cholinesterase as predictors of diabetic neuropathy have not been indicated previously. The renal injury-related factors (urinary microalbumin, urinary Cr, Ca, crystallization, and P) identified as diabetes neuropathy and peripheral vascular disease predictors were rarely identified in diabetic medical research. Thus, our study simultaneously suggests the correlation between nephropathy and neuropathy/ peripheral vascular disease. Besides, the predictors of retinopathy such as phosphocreatine kinase CPK, creatine kinase-MB mass, urinary microalbumin, and urinary microalbumin creatinine demonstrated a correlation between retinal vascular changes and heart [26, 27]renal injury [29, 30]. These results show the efficiency of our model for disease prevention and medical research assistance.

There are still several limitations to this study. First, this model is designed for diagnose diabetes and complications. It is disease nature that limits its application in non-diabetic patients. Second, we used large amounts of data, and most factors become statistically significant but clinically irrelevant. To impact clinical usage, medical knowledge and related researches are needed. Finally, as we assembled the data only from one hospital, this action limits the generalizability of our model to other group of people who are distinguished by some other essential predictors. Our primary intention was just to demonstrate the feasibility of the MLC model. Further investigation on multi-center study is in our horizon.

## Conclusion

In this study, we demonstrated the correlations between different diabetic complications from two perspectives: statistical analysis (PCC) and machine learning (MLC). We illustrated that the MLC model is effective to leverage these correlations and outperforms the traditional binary classification model in predicting diabetic complications. This study is essential not only because we provided a better model in predicting diabetic complications but also because our model could be adapted and applied to leverage correlations among complications and predict outcomes in other complex diseases. In the future, we intend to extend our method to other diseases.

## Supplementary Information

**Additional file 1: Table 1**. 93 clinical parameters collected from EHR. **Table 2**. Qualitative data assignments. **Table 3**. Experimental results on 5 different models including 11 performance evaluation metrics, taking XGBoost as the base model. **Fig. 1**. The ROC curves using XGBoost as the base model. The experimental results of 4 different models, BR, LP, CC, and ECC. In each figure, the ROC curves of different complications are marked by different colors accordingly.

## Data Availability

The datasets generated and analyzed during the current study are not publicly available due to data privacy laws, but are available from the corresponding author on reasonable request.

## Abbreviations

T2DM: Type 2diabetes mellitus
EHR: Electronic health record
MLC: Multi-label classification
ALT: Alanine aminotransferase
AST: Aspartate aminotransferase
GGT: R glutamyl transferase
Cr: Creatinine
mAlb: Microalbumin
CPK: Creatine phosphokinase
ESR: Erythrocyte sedimentation rate
CK-MB: Creatine kinase-MB
INR: PT international standardized ratio
ACR: Albumin-to-creatinine ratio
BUN: Blood urea nitrogen
Ca: Calcium
P: Phosphorus
RBC: Red blood cell
Apo A1: Apolipoprotein A1
Apo B: Apolipoprotein B
USECs: Urinary Squamous Epithelial Cells
LP: Label Power Set
CC: Classifier Chains
ECC: Ensemble Classifier Chains
CLR: Calibrated Label Ranking
BR: Binary Relevance
