# Supplementary material for: Application of multi-label classification models for the diagnosis of diabetic complications
Source: BMC Med Inform Decis Mak. 2021 Jun 7;21:182. doi: 10.1186/s12911-021-01525-7 (PMC8182940; doi:10.1186/s12911-021-01525-7)
Supplement: Supplementary file 1 — Additional file 1: Table 1. 93 clinical parameters collected from EHR. Table 2. Qualitative data assignments. Table 3. Experimental results on 5 different models including 11 performance evaluation metrics, taking XGBoost as the base model. Fig. 1. The ROC curves using XGBoost as the base model. The experimental results of 4 different models, BR, LP, CC, and ECC. In each figure, the ROC curves of different complications are marked by different colors accordingly. [file 12911_2021_1525_MOESM1_ESM.doc]

**Additional file**

**Title page**

**Title：**Application of multi-label classification models for the diagnosis of diabetic complications

**Authors and affiliations**

Liang Zhou^1#^**,** Xiaoyuan Zheng^1#^, Di Yang^2^, Ying Wang^1^, Xuesong Bai^3^, Xinhua Ye^1*^

1. Department of Endocrinology, The Affiliated Changzhou No.2 People's Hospital of Nanjing Medical University, 29 Xinglongxiang Road, Changzhou City, Jiangsu Province, 213000, China

2. Shanghai Jiao Tong University School of Medicine, Shanghai, 200025,China

3. Capital Medical University, Beijing 100053,China

# Liang Zhou, Xiaoyuan Zheng contributed equally and are co-first authors.

**Corresponding author**

Xinhua Ye [czyxh2000@163.com](mailto:czyxh2000@163.com)

Department of Endocrinology, The Affiliated Changzhou No.2 People's Hospital of Nanjing Medical University, 29 Xinglongxiang Road, Changzhou City, Jiangsu Province, 213000, China

**Additional Table 1**

93 clinical parameters collected from EHR

| **laboratory data** |  |
| --- | --- |
|  | Age |
|  | Gender |
|  | Systolic / diastolic blood pressure |
|  | BMI |
| **Urine** |  |
|  | Bilirubin |
|  | Urobilinogen |
|  | Ketones |
|  | Urine occult blood |
|  | Protein |
|  | Nitrite |
|  | Urine white blood cells |
|  | Urine glucose |
|  | Specific gravity |
|  | PH value |
|  | Vitamin C |
|  | Urine calcium |
|  | Urine microalbumin |
|  | Urine albumin to creatinine ratio (ACR) |
|  | urinary microalbumin/creatinine |
|  | Crystal |
|  | Clear cast |
|  | Squamous epithelial cells |
|  | Renal tubular epithelial cells |
|  | Bacteria |
|  | Urinary trace albumin |
|  | creatinine |
|  | hydroxyl vitamin |
|  | urinary protein quantitative |
|  | Urinary albumin-creatinin ration |
| **Blood** | Total bilirubin |
|  | direct bilirubin |
|  | total protein |
|  | albumin |
|  | globulin |
|  | alanine aminotransferase (ALT) |
|  | Aspartate Aminotransferase (AST) |
|  | alkaline phosphatase (ALP) |
|  | R glutamine transferase (GGT) |
|  | lactic acid dehydrogenase |
|  | choline lipase |
|  | total bile acid |
|  | adenosine deaminase |
|  | glucose |
|  | Uric acid |
|  | Blood Urea nitrogen (BUN) |
|  | Serum creatinine (Cr) |
|  | total cholesterol |
|  | triglycerides |
|  | HDL Cholesterol |
|  | LDL Cholesterol |
|  | Apolipoprotein A1 (Apo A1) |
|  | Apolipoprotein B (Apo B) |
|  | Apolipoprotein A (Apo A) |
|  | Phosphoric acid kinase |
|  | Creatine phosphate kinase (CK) |
|  | Potassium (K) |
|  | Sodium (Na) |
|  | Chlorine （Cl） |
|  | Total carbon dioxide （CO2） |
|  | Calcium (Ca) |
|  | Phosphorus (P) |
|  | serum C peptide determination (60min) |
|  | serum C peptide determination (120min) |
|  | Fasting insulin |
|  | insulin (120min) |
|  | insulin (60min) |
|  | Glycated hemoglobin |
|  | Blood sugar after meals |
|  | white blood cells |
|  | Neutrocyte Absolute Value |
|  | Lymphocyte Absolute Value |
|  | Monocytes Absolute |
|  | Eosinophils |
|  | Neutrocyte Percentage |
|  | Lymphocyte Percentage |
|  | Monocyte Percentage |
|  | Eosinophil Percentage |
|  | Red Blood Cell (RBC) |
|  | Hemoglobin (Hb) |
|  | Hematocrit |
|  | Mean Corpuscular Volume |
|  | Mean hemoglobin content |
|  | Mean hemoglobin concentration |
|  | Erythrocyte distribution width CV |
|  | Platelet |
|  | mean platelet volume |
|  | Platelet plenum |
|  | platelet distribution width |
|  | large platelet ratio |
|  | Prothrombin time |
|  | PT international standardized ratio |
|  | activated partial thromboplastin time |
|  | Antithrombin time III |
|  | thrombin time |
|  | D-dimer |
|  | erythrocyte sedimentation rate (ESR) |
|  |  |

**Additional Table 2. Qualitative data assignments**

| Indicators | Value | Quantified as |
| --- | --- | --- |
| Sex | Female/male | 0/1 |
| Urinogen, bilirubin, ketone body, urine occult blood, protein, nitrite, urine white blood cell, urine sugar, and urine tube type | normal and negative (-)/1+, 2+ | 0/1, 2 |
| Urinary microalbumin creatinine ratio (ACR) | <3.4/ 3.4-33.9/ >33.9 | 0/1/2 |
| D dimer | <0.1/ >40 | 0.1/40 |
| Urinary trace albumin | <5.0/ >300 | 5.0/ 300 |
| Urinary creatinine | <15.0/<16.4 | 15.0/16.4 |
| Missing value filling part | <n/>m | n/m |

**Additional Table 3. Experimental results on 5 different models including 11 performance evaluation metrics, taking XGBoost as the base model**

| **Metric** | Traditional model | The MLC models | | | |
| --- | --- | --- | --- | --- | --- |
|  | **BR** | **LP** | **CC** | **ECC** | **CLR** |
| **Example-based Metrics** |  |  |  |  |  |
| Hamming loss | 0.1739 | 0.1769 | 0.1738 | **0.1721** | 0.1723 |
| Accuracy | 0.6928 | 0.6896 | 0.6967 | **0.7033** | 0.6924 |
| F1_score | 0.7787 | 0.771 | 0.7787 | **0.7869** | 0.7772 |
| Precision | 0.8364 | 0.8102 | 0.8373 | **0.8657** | 0.8284 |
| Recall | 0.7859 | **0.8002** | 0.7847 | 0.7753 | 0.7927 |
| **Label-based Metrics** |  |  |  |  |  |
| F1_micro | 0.8033 | 0.7963 | 0.8048 | **0.8115** | 0.8031 |
| F1_macro | 0.7647 | 0.7586 | **0.7705** | 0.7688 | 0.7699 |
| Precision_micro | 0.7792 | 0.7857 | 0.7753 | 0.7644 | **0.7867** |
| Precision_macro | 0.8017 | 0.7799 | 0.8007 | 0.7427 | **0.8102** |
| Recall_micro | 0.8289 | 0.8072 | 0.8366 | **0.8647** | 0.8202 |
| Recall_macro | 0.7492 | 0.7473 | 0.7616 | **0.8011** | 0.7482 |


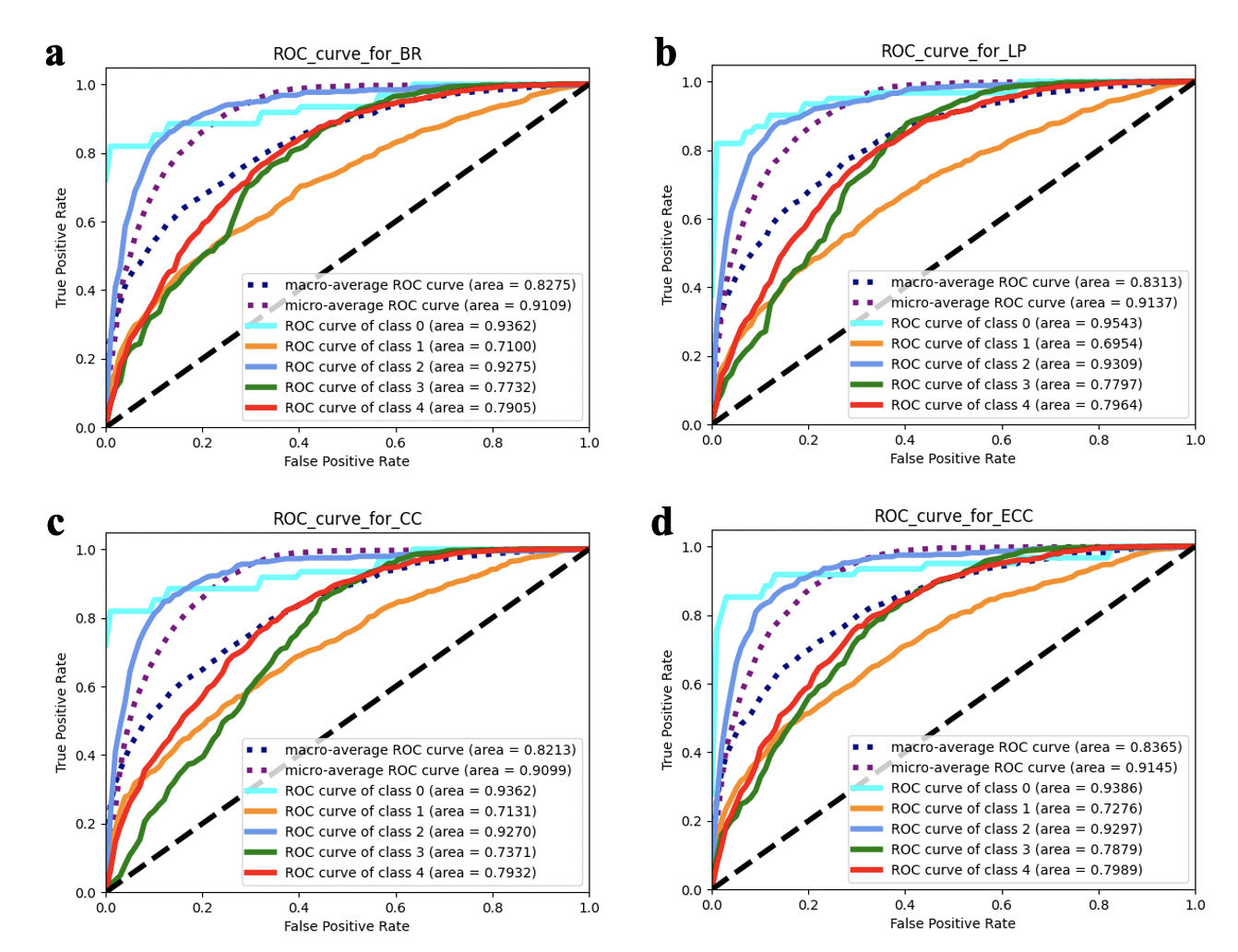


**Additional Fig.1** **The ROC curves using XGBoost as the base model.** The experimental results of 4 different models, BR, LP, CC, and ECC. In each figure, the ROC curves of different complications are marked by different colors accordingly.
